# Healthcare-associated HIV outbreaks in Pakistan: a preventable public health crisis

**DOI:** 10.5588/pha.26.0032

**Published:** 2026-05-18

**Authors:** F. Malik

**Affiliations:** University Institute of Public Health, The University of Lahore, Lahore, Pakistan.

**Keywords:** iatrogenic infection, syringe reuse, intravenous injections, infection prevention

Dear Editor,

In November 2025, a suspected paediatric HIV outbreak was reported at the Kulsum Bai Valika Social Security Hospital in SITE Town, Karachi.^1^ Several children were found to be HIV positive after receiving care at the hospital, prompting an investigation by provincial authorities and renewed public alarm. This event is not an isolated one. It is the latest manifestation of a troubling pattern: repeated, preventable, healthcare-associated HIV outbreaks occurring across Pakistan. The concentration of these outbreaks among young children, often living in communities where conventional risk factors for HIV are absent, points to a systemic public health crisis. The recent outbreak in Karachi demands that we confront the uncomfortable truth: Pakistan’s HIV outbreaks are driven by health-system failures.^2,3^

The epidemiological features of the Karachi cluster closely mirror those of earlier outbreaks. In 2019, the Ratodero outbreak in Larkana district remains the most widely documented example. A total of 876 people, most of them children under 15 years of age, were diagnosed HIV positive during mass screening campaigns.^4^ Investigations by the WHO concluded that widespread syringe reuse, unsafe intravenous injections, poor infection prevention and control practices, and unregulated medical procedures were central drivers. Many of the affected children had HIV negative parents, ruling out vertical transmission and strongly indicating iatrogenic exposure. Earlier, from 2018 to 2019, an outbreak in Kot Imrana in Sargodha district revealed more than 800 HIV positive individuals in a village of about 5,000 residents.^5^ Prevalence increased from an initial level of around 1% to beyond 13%, and the increase was traced to the practices of a single unqualified healthcare provider who reused syringes across patients. The Kot Imrana investigation provided one of the clearest examples of how informal healthcare providers can initiate large, sustained chains of transmission through unsafe injections.

Iatrogenic HIV spread has not been limited to the informal sector. In 2016, an outbreak among haemodialysis patients at Chandka Medical College Hospital in Larkana demonstrated that tertiary care institutions are also vulnerable.^6^ The event revealed lapses in sterilization, equipment handling, and compliance with basic infection control protocols. In 2024, a similar pattern was observed at the Nishtar Hospital dialysis unit in Multan where 30 patients tested HIV positive after receiving treatment.^7^ These hospital based outbreaks show that unsafe clinical environments are not confined to small unregulated clinics and that even large public hospitals remain susceptible to systemic IPC failures.

More recently, paediatric outbreaks have re-emerged. In 2024, provincial records from Mirpurkhas documented more than 150 HIV positive children, representing more than one quarter of all paediatric HIV notifications in Sindh for that year.^8^ The epidemiologic profile strongly suggests unsafe injection practices and unregulated community healthcare. Before the Karachi cluster,^1^ the most alarming event occurred in Taunsa Sharif in Dera Ghazi Khan district, where between late 2024 to mid-2025, nearly 300 children (the majority under five years of age), were found to be HIV positive.^9^ A joint WHO–UNICEF assessment identified widespread unsafe injections, unlicensed clinics, and gaps in infection prevention within both formal and informal care. These outbreaks illustrate a national pattern of preventable iatrogenic HIV transmission. They are not isolated incidents but symptoms of deep-seated health system dysfunction.^2^

Pakistan continues to experience these outbreaks because unsafe medical practices persist across both unregulated and formal sectors. Each year, millions of patient contacts occur with unlicensed practitioners who frequently rely on injections, reuse syringes to reduce cost, and operate without oversight. Multiple outbreak investigations in Ratodero, Kot Imrana, and Taunsa have directly implicated such providers.^2,4^ Pakistan also has one of the highest injection rates per capita globally. There is a widespread perception that injectable therapy is inherently more effective, which drives patient demand and encourages providers to administer injections even when unnecessary. This creates conditions where sterility cannot be reliably ensured. At the same time, tertiary hospitals have their own vulnerabilities. The Larkana and Multan dialysis outbreaks showed that even institutions with specialist services may have lapses in sterilization, equipment reuse, and incomplete adherence to IPC standards.^6,7^ Audit mechanisms remain infrequent and enforcement inconsistent across provinces. Structural inequities further aggravate the problem. Rural poverty and limited access to qualified providers push communities toward informal practitioners and amplify their exposure to unsafe medical procedures.

Preventing future outbreaks requires a coordinated response that addresses regulatory, clinical, and community level drivers. A national licensing and accreditation system for all healthcare providers must be implemented and enforced. Although provincial authorities have recently moved to close unlicensed clinics, these actions must be sustained and transparent. Large scale public health communication is also necessary. Pakistan needs a campaign comparable to polio eradication efforts to reduce the demand for unnecessary injections, promote oral alternatives, and educate communities about the dangers of syringe reuse. Infection prevention and control must be strengthened through routine audits of all facilities and the linking of audit results to certification, penalties, and retraining. The national policy mandating auto disable syringes must be applied universally with strict supply chain monitoring to prevent counterfeit or previously used syringes from returning to circulation. Blood banks and transfusion centres require accreditation, external quality assurance, and consistent screening for HIV, hepatitis B and C. Community empowerment is essential, and patients must be supported to recognise safe medical practice, refuse reused syringes, and report unregistered providers.

The 2025 Karachi paediatric HIV outbreak is not an anomaly. It is the most recent link in a chain of predictable and preventable outbreaks driven by structural weaknesses in Pakistan’s health system. With less than four years to go until the Sustainable Development Goals targets, the country faces a decisive question. It can either continue reacting to crises or commit to reforms that address the roots of iatrogenic HIV transmission. The clinical tools necessary to end these outbreaks already exist. What is needed is the political will to build a healthcare environment in which no one acquires HIV from the very system meant to heal them.
